# Hemosuccus Pancreaticus: A Challenging Diagnosis and Complex Management

**DOI:** 10.7759/cureus.86403

**Published:** 2025-06-19

**Authors:** Ahmed Hassanien, Abdullah Motam

**Affiliations:** 1 Internal Medicine, East Lancashire University Hospitals NHS Trust, Blackburn, GBR; 2 Gastroenterology, Royal Blackburn Teaching Hospital, Blackburn, GBR

**Keywords:** endoscopic ultrasound (eus), endoscopy, hemosuccus pancreaticus, pancreatitis, pseudoaneurysm, upper gastrointestinal bleeding

## Abstract

Hemosuccus pancreaticus (HP) is an uncommon and infrequently reported cause of upper gastrointestinal bleeding. It is defined as a hemorrhage into the pancreatic duct, which subsequently drains into the duodenum via the ampulla of Vater. The condition is typically associated with underlying pancreatic pathology, such as pancreatitis, pseudocysts, or vascular abnormalities, including splenic artery aneurysms. Clinically, HP presents with intermittent gastrointestinal bleeding, epigastric pain, and a drop in hemoglobin levels; features that often delay diagnosis due to their non-specific and episodic nature. Early recognition is essential, as the condition carries a high risk of morbidity and mortality if not promptly managed. We present a diagnostically challenging case of HP in a frail patient with multiple comorbidities, highlighting the importance of clinical vigilance and multidisciplinary coordination.

## Introduction

Hemosuccus pancreaticus (HP) is defined as a hemorrhage originating from the ampulla of Vater via the pancreatic duct into the second portion of the duodenum [1]. It represents a rare etiology of upper gastrointestinal bleeding, accounting for fewer than 1% of such cases, with an incidence of approximately one in 1,500 patients presenting with upper gastrointestinal hemorrhage [2]. The condition demonstrates a marked male predominance, with a male-to-female ratio of 7:1, and predominantly affects individuals in their fifth to sixth decades of life. The mortality rate in untreated cases can be as high as 90%, whereas, with appropriate intervention, it decreases to between 25% and 37% [3].

Lower and Farrell documented the initial report of HP in 1931 [4], while the term “hemosuccus pancreaticus” was introduced, along with a detailed clinical description, by Philip Sandholm in 1970 [5]. The most frequently reported cause of HP is pancreatic inflammation, encompassing acute, chronic, and hereditary pancreatitis. Inflammatory processes within the pancreatic ductal system contribute to the disruption and eventual rupture of adjacent vascular structures [4].

Additional contributory factors include intraductal gallstones and the development of pancreatic pseudocysts. Of particular significance, pancreatic pseudocysts may secrete proteolytic enzymes such as elastase, which degrade the elastic lamina of neighboring vessels, thereby predisposing them to hemorrhage [6]. Visceral arterial aneurysms located within or adjacent to the pancreas constitute another important etiology implicated in approximately 6-17% of HP cases [4]. The splenic artery is most commonly involved; however, aneurysms of the hepatic, gastroduodenal, and pancreatoduodenal arteries have also been reported [7]. Pancreatic neoplasms, both benign entities, such as serous or mucinous cystic neoplasms, and malignant tumors, including pancreatic adenocarcinoma and neuroendocrine tumors, have been associated with HP. Rarely, metastatic lesions, notably from renal cell carcinoma, have been implicated. The mechanism underlying hemorrhage involves tumor invasion into adjacent vascular or ductal structures, facilitating bleeding into the pancreatic ductal system [8].

## Case presentation

A 69-year-old man presented with melena and symptoms of anemia, including dyspnea and dizziness. His past medical history included chronic alcoholic pancreatitis, alcoholic fatty liver disease, hypertension, chronic obstructive pulmonary disease, and osteoporosis. He was suspected of having an acute upper gastrointestinal hemorrhage. Risk stratification using the Glasgow-Blatchford Score (GBS) yielded a score of 14, indicating that urgent endoscopy was required. The GBS is utilized to assess patients with suspected upper gastrointestinal bleeding [9]. A score of 14 suggests a high risk that necessitates prompt intervention. This score was calculated by considering several parameters (Table 1).

**Table 1:** Glasgow-Blatchford Score calculation of 14, with relevant parameters.

Parameter	Value	Score
Hemoglobin	63 g/L	6
Urea	13.7 mmol/L	4
Systolic blood pressure	100 mmHg	1
Melena presence	Yes	1
History of hepatic disease	Yes	2
Total score	14	

**Figure 1:**
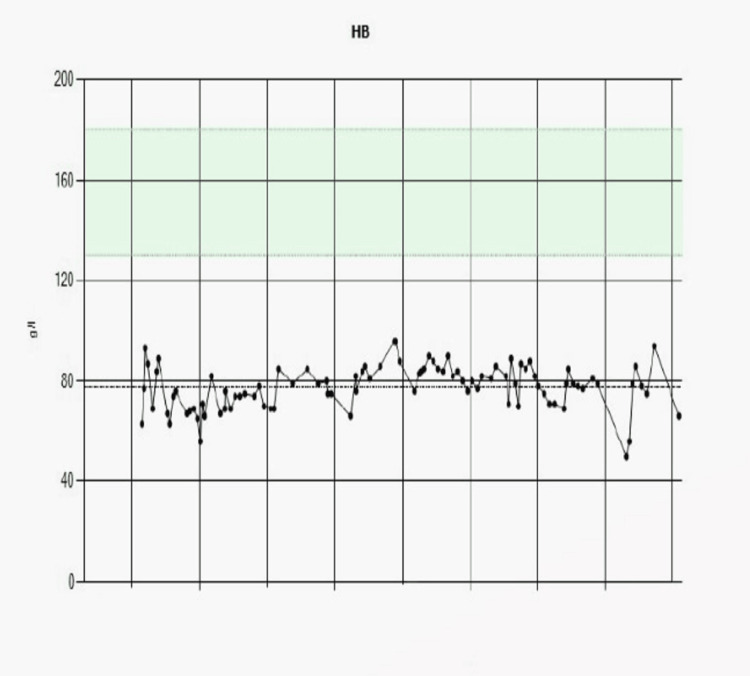
Persistently low hemoglobin levels over time, with values consistently below the normal reference range of approximately 120–160 g/L. Most readings fall between 60 and 90 g/L, indicating chronic anemia. There is some fluctuation in the values, including occasional dips below 60 g/L, suggesting intermittent worsening.

Following stabilization with intravenous fluids and blood transfusion, the patient underwent esophagogastroduodenoscopy (EGD), which revealed grade C esophagitis, with features suggestive of concomitant *Candida* infection. High-dose proton pump inhibitor therapy and oral antifungal treatment were initiated, with a plan to repeat endoscopy after six weeks to assess mucosal healing. However, these findings did not fully explain the ongoing decline in hemoglobin despite repeated transfusions and persistent melena (Figure 1).

**Figure 2:**
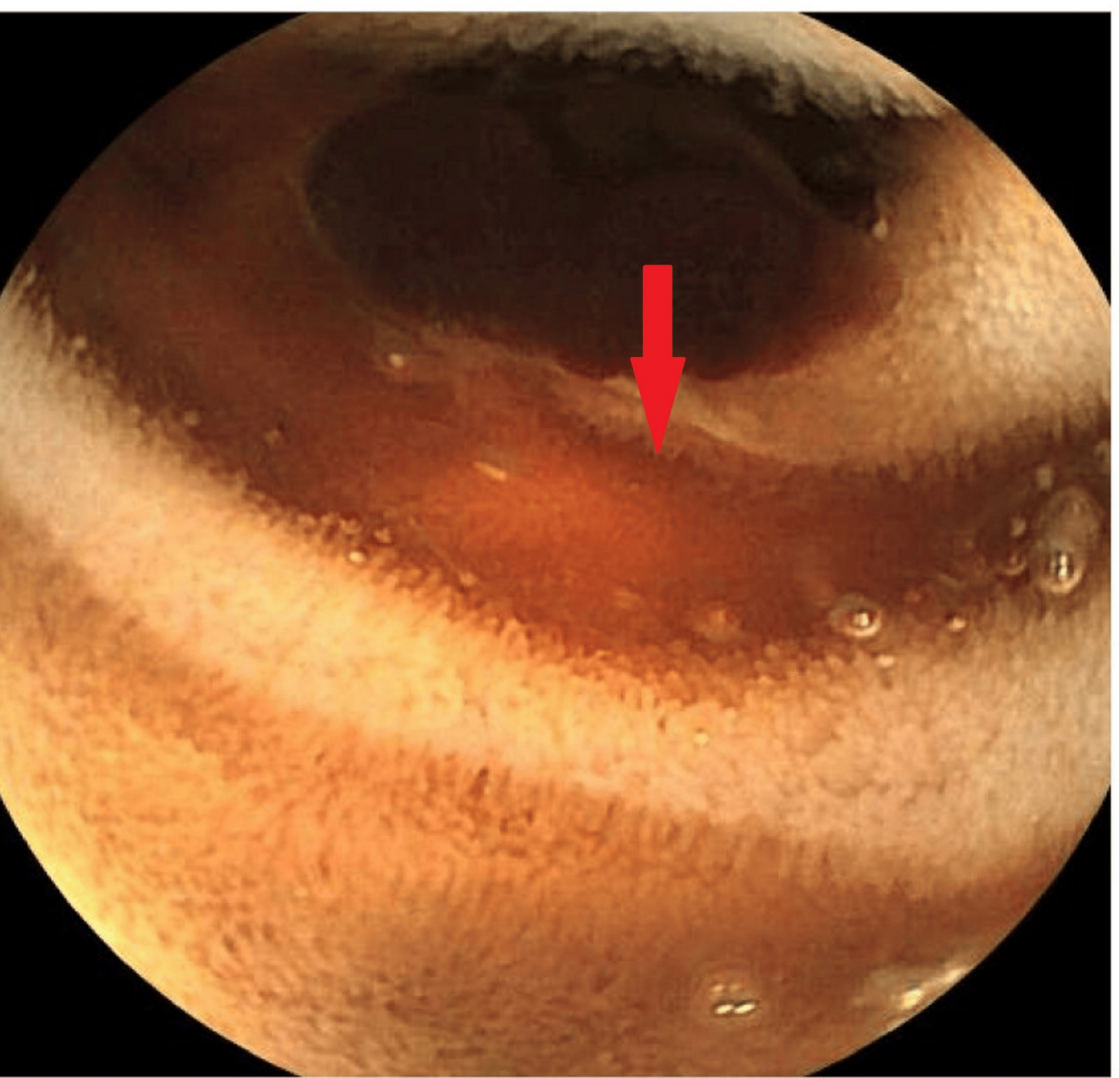
Capsule endoscopy revealing a large intraluminal clot within the small intestine (red arrow), consistent with recent gastrointestinal bleeding.

Due to the ongoing melena of unclear origin, a CT angiogram of the abdomen was performed, which revealed no arterial blush or contrast extravasation and, thus, no evidence of active gastrointestinal bleeding. The decision was made to proceed with video capsule endoscopy, which identified active, slow bleeding localized to the early to mid-jejunum (Figure 2).

**Figure 3:**
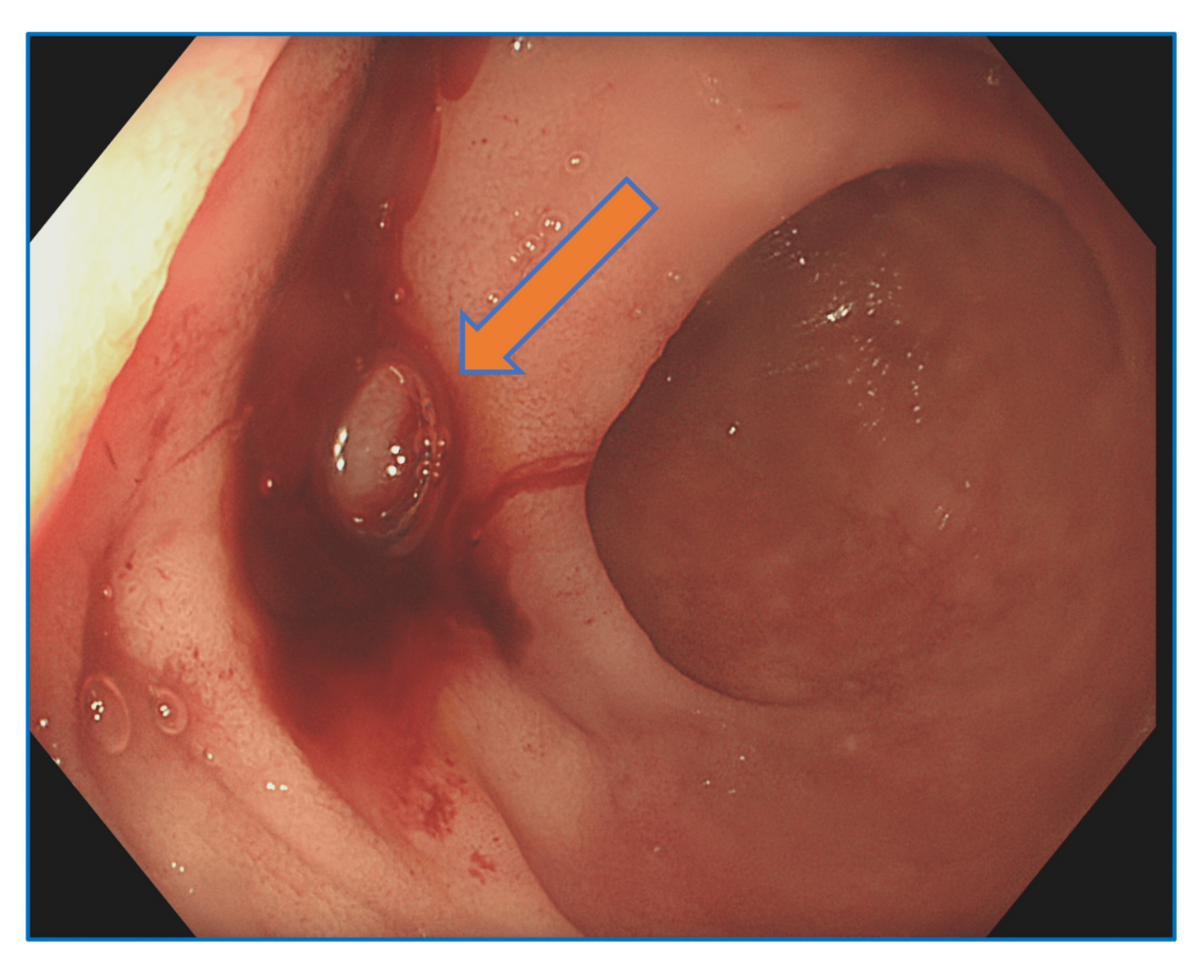
Esophagogastroduodenoscopy showing bleeding spots near the ampulla of Vater area.

**Figure 4:**
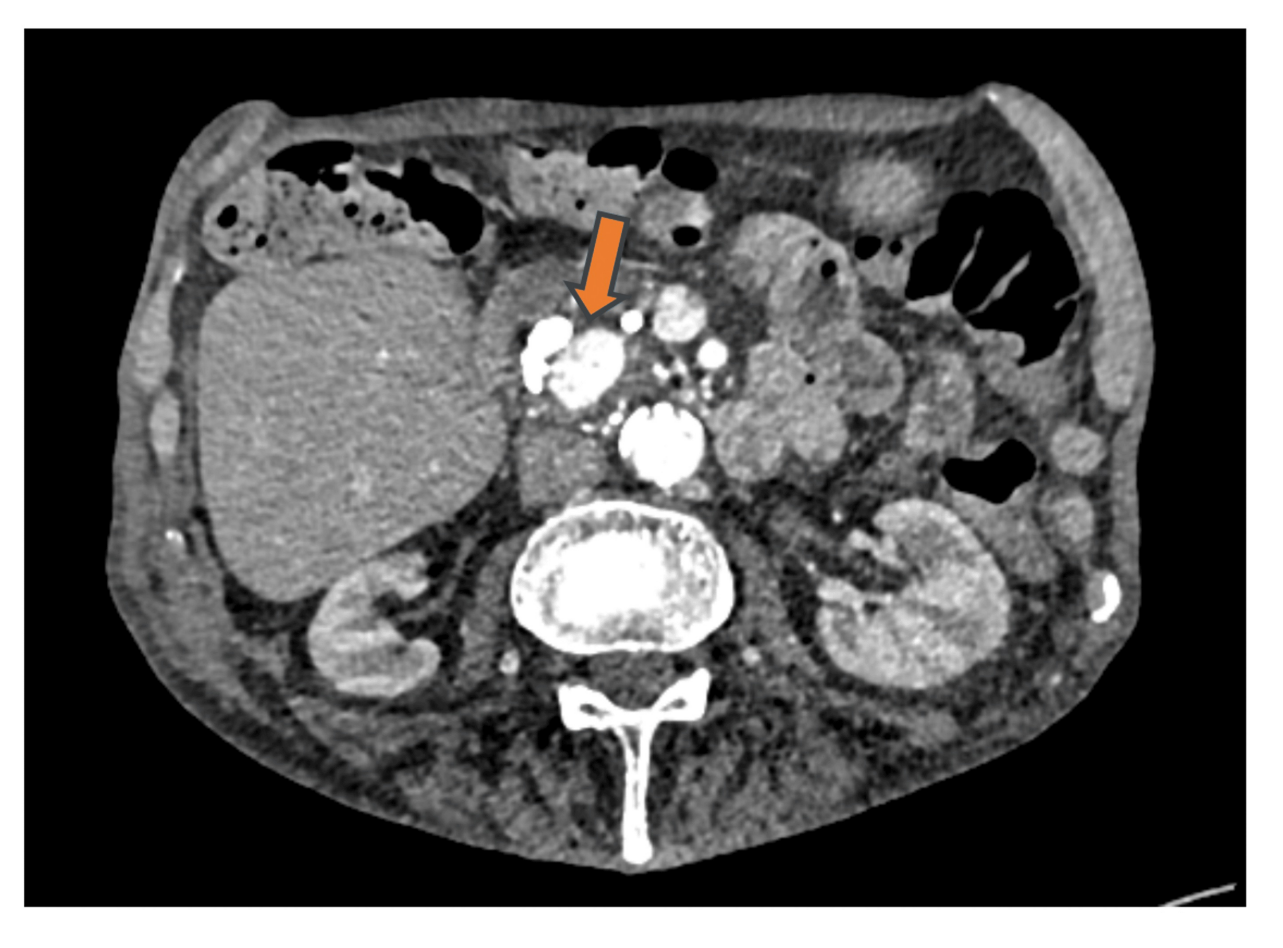
Contrast-enhanced CT scan of the abdomen showing a 3.3 cm × 1.4 cm × 2.2 cm aneurysm involving the head and uncinate process of the pancreas.

Suspecting that the source of bleeding was enteric, an anterograde enteroscopy was performed, which revealed active bleeding near the ampulla of Vater (Figure 3). This finding raised concerns about the possibility of a pancreatic-arterial fistula as the cause of the hemorrhage. Consequently, additional imaging was recommended. Triple-phase contrast-enhanced CT of the liver revealed a 3.3 × 1.4 × 2.2 cm aneurysm located in the pancreatic head and uncinate process (Figure 4).

The patient was referred to interventional radiology for conventional angiography and possible selective embolization. Despite catheterization of the superior mesenteric artery and celiac axis using a microcatheter, no definitive arterial source of bleeding was identified.

**Figure 5:**
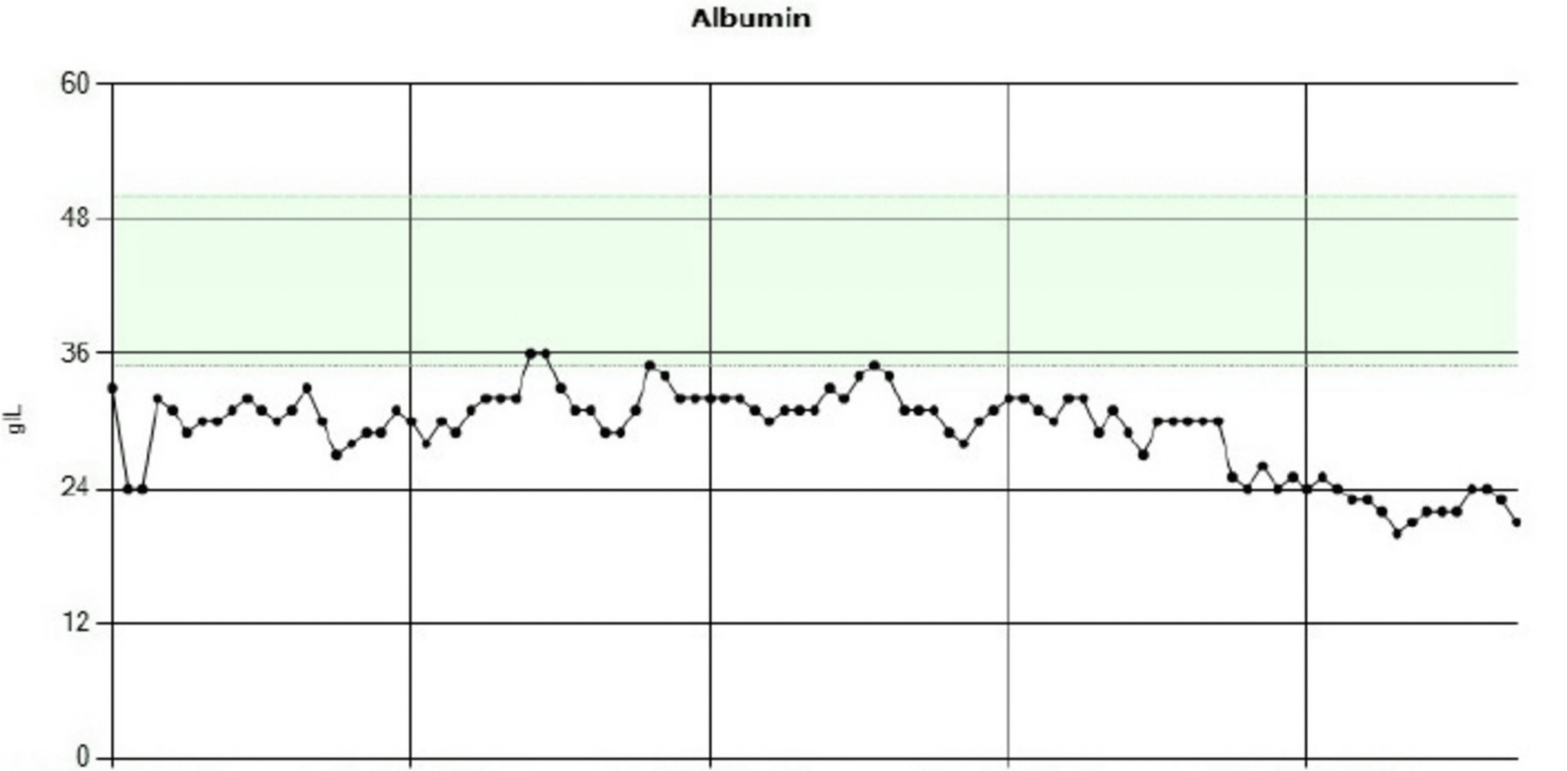
Serial serum albumin measurements showing persistent hypoalbuminemia with a progressive downward trend. Values consistently remained below the normal reference range (35-50 g/L), with a marked decline in the later stages, indicative of worsening nutritional status and chronic inflammation.

A multidisciplinary team meeting recommended an endoscopic ultrasound (EUS)-guided intervention targeting the distal pancreas near the ampulla, recognizing the low probability of success. If unsuccessful, empirical embolization of the inferior pancreaticoduodenal artery was to be considered. Surgical intervention was deemed high risk due to the patient’s poor nutritional status, evidenced by persistently low serum albumin levels (Figure 5) and a World Health Organization Performance Status (WHO-PS) score of 3.

**Figure 6:**
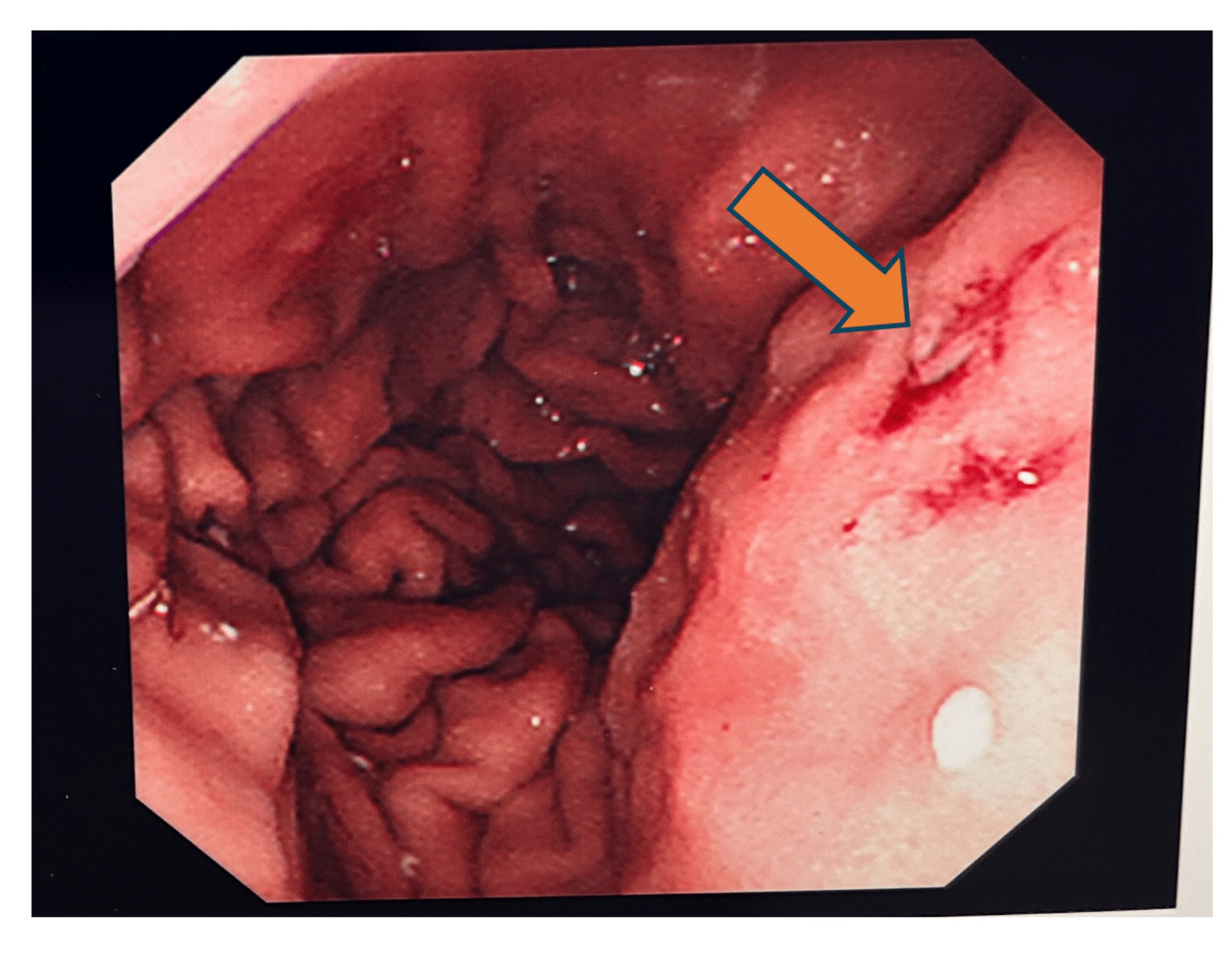
Endoscopic view of bleeding spots near the ampulla of Vater area.

**Figure 7:**
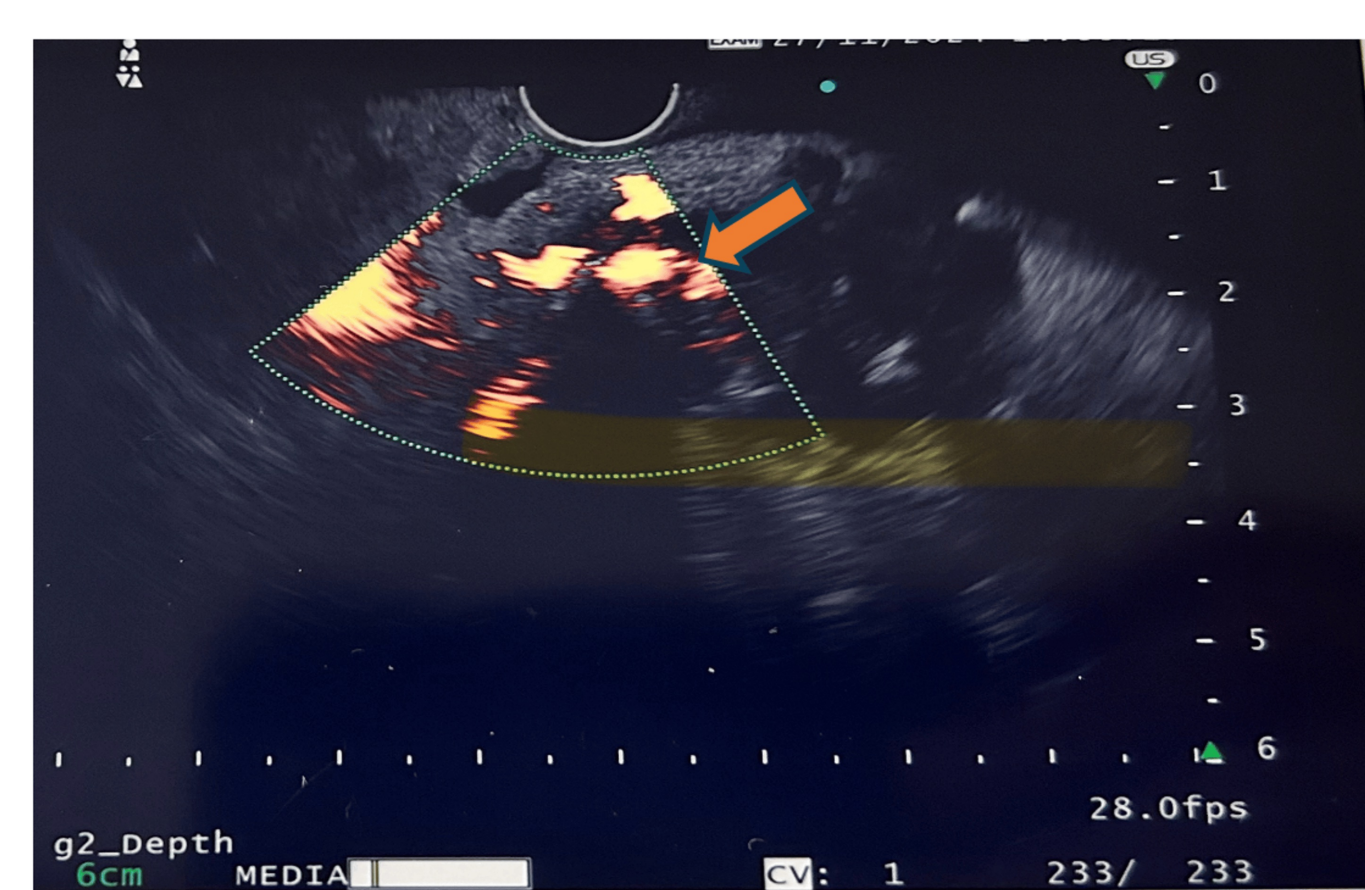
Color Doppler imaging showing blood flow inside the pseudoaneurysm.

**Figure 8:**
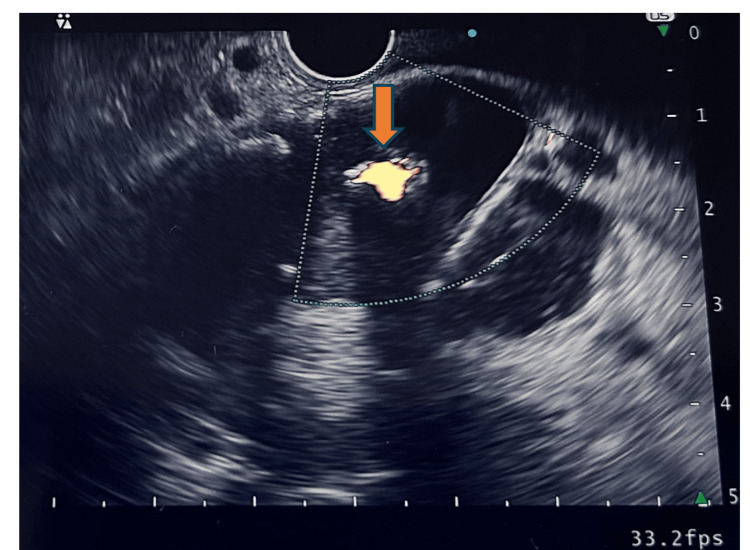
Color Doppler imaging showing blood flow inside the pseudoaneurysm.

During a subsequent EUS procedure, fresh blood was observed at a bulging ampulla (Figure 6). Imaging further revealed necrotic pancreatic parenchyma with peripheral calcifications and a 21 × 14 mm aneurysm with a feeding vessel in the submucosa of the second part of the duodenum, adjacent to the ampulla (Figures 7, 8). Approximately 4.5-5 mL of diluted thrombin (500 U/mL, 1:1 with normal saline) was injected into the aneurysm, resulting in immediate cessation of blood flow.

The patient initially demonstrated hemodynamic and hematologic stability over the subsequent two days. However, he subsequently experienced massive melena with a significant hemoglobin decline from 110 g/L to 67 g/L, resulting in hemorrhagic shock (blood pressure, 84/56 mmHg; heart rate, 126 beats/minute; respiratory rate, 22 breaths/minute; SpO_2_, 92% on 35% Venturi mask) and lactic acidosis (lactate 5.9 mmol/L). Despite aggressive resuscitative efforts, the patient’s clinical status deteriorated, culminating in death.

## Discussion

HP is a rare and potentially life-threatening obscure cause of upper gastrointestinal bleeding. It is described as bleeding from the ampulla of Vater via the pancreatic duct into the second part of the duodenum (D2) [1,2]. This bleeding originates from a source in the pancreas, pancreatic duct, or adjacent structures, such as the splenic or gastric arteries, which can communicate with the pancreatic duct and cause intraductal bleeding [4].

In terms of etiology, it is well-reported that the leading cause of HP is chronic pancreatitis, particularly pseudoaneurysm formation in peripancreatic vessels [10]. However, in some rare cases, it can be due to acute pancreatitis, pancreatic tumors, aneurysmal rupture, ductal calculi, arteriovenous malformations, trauma, and iatrogenic causes, amongst others.

Clinically, patients often present with upper gastrointestinal bleeding, predominantly melena, which tends to be intermittent and of variable severity. A heightened clinical suspicion of HP is warranted in individuals exhibiting the triad of abdominal pain, gastrointestinal hemorrhage, and elevated serum amylase, particularly in the setting of chronic pancreatitis, pancreatic neoplasms, or pancreatic vascular abnormalities [2].

The diagnosis of HP remains a clinical challenge due to its rarity, anatomical complexity, and intermittent symptomatology [2]. Abdominal pain is thought to be caused by increased intraductal pressure due to distension of the pancreatic duct with blood. Bleeding invariably follows thereafter, within the next day or two, relieving the pain, as the pancreatic duct is decompressed and blood leaves the pancreas via the ampulla of Vater [2].

Laboratory investigations are non-diagnostic, with biochemical markers primarily reflecting blood loss. Serum amylase levels can be raised in the context of acute pancreatitis or from HP itself due to increased intraductal/intracystic pressure [2,11].

The mainstay of diagnosis is through imaging and endoscopy. EGD is the first-line investigation, primarily essential to exclude other causes of gastrointestinal bleeding, such as varices, ulcers, and erosive gastritis, and in approximately 30% of cases, active bleeding from the duodenal ampulla may be directly visualized [12-14], as was observed in our patient.

Unfortunately, in most cases, EGD is insufficient due to the forward-viewing gastroscope and the intermittent nature of bleeding. Ultrasonography, particularly Doppler or dynamic techniques, can assist in identifying pancreatic pseudocysts or aneurysms of peripancreatic vessels and has been reported as a useful diagnostic tool [14]. Likewise, contrast-enhanced CT is an excellent modality for detecting pancreatic pathology, including features of chronic pancreatitis, pseudocysts, and pseudoaneurysms [15].

A definitive diagnosis typically relies on angiography, which remains the gold standard for diagnosis [2]. Angiography delineates the vascular anatomy, identifies the causative artery, and facilitates simultaneous therapeutic intervention. Selective catheterization of the celiac trunk and superior mesenteric artery enables visualization of the main pancreatic duct through contrast opacification, confirming the presence of HP. Angiography also detects aneurysms or pseudoaneurysms, with a sensitivity approaching 96% [2,16].

Approaches to the management of HP should focus on hemodynamic stabilization, aggressive resuscitation protocols, and bleeding source eradication. There are two potential approaches, namely, surgery and interventional radiology via angiographic embolization. Interventional radiology is favored as the initial treatment modality, demonstrating immediate success rates ranging from 60% to 100% [17]. Nonetheless, ischemia may occur in the vascular territory supplied by the embolized vessel if collateral circulation is inadequate. Consequently, embolization of major arteries, such as the celiac trunk, common hepatic artery, or superior mesenteric artery, is contraindicated. Additional complications include aneurysm infection and splenic infarction [11].

Surgical intervention is reserved for cases of uncontrolled hemorrhage, persistent shock, embolization failure, or when embolization is contraindicated. Surgery is also indicated in patients requiring operative management of concomitant complications, including pseudocysts, pancreatic abscesses, gastric outlet obstruction, or intractable pain [18]. Surgical options include distal pancreatectomy with splenectomy, central pancreatectomy, intracystic ligation of the blood vessel, aneurysm ligation, and bypass graft [2]. Most surgical procedures have shown success rates of 70-85%, but at the same time, operative mortality rates of 10-50% have been reported in the literature [2]. The rate of rebleeding after surgery is around 0-5% [2].

Recently, EUS-guided angiotherapy has emerged as a novel therapeutic technique. Will et al. first described the successful treatment of HP using the ultrasound-guided transcutaneous application of fibrin glue and histoacryl injection [19,20].

In our case, angiographic evaluation did not reveal a definitive source of hemorrhage, and surgical intervention was considered high risk in light of the patient’s poor nutritional status and low WHO-PS score. Given the limitations of conventional therapies in our case, EUS-guided intervention represented the most feasible option. Ultimately, EUS-guided thrombin injection resulted in the initial cessation of bleeding; however, hemorrhage recurred and ultimately became refractory to management, leading to death.

## Conclusions

HP is a rare but potentially life-threatening condition that requires a high degree of clinical suspicion, particularly in patients with chronic pancreatitis. Diagnosis is often complex, and management can be equally challenging. Even after interventional radiology embolization, there is a risk of recurrence, and surgical intervention carries its own set of complications. EUS has emerged as a promising therapeutic approach, allowing for ultrasound-guided transcutaneous injection of fibrin glue, histoacryl/lipiodol, or thrombin, which has shown success in achieving hemostasis. Nevertheless, the risk of recurrence with life-threatening bleeding remains a major concern.
